# Comprehensive Analysis of NKX3.2 in Liver Hepatocellular Carcinoma by Bigdata

**DOI:** 10.3390/medicina59101782

**Published:** 2023-10-06

**Authors:** An-Na Bae, Jongwan Kim, Jong-Ho Park, Jae-Ho Lee, Euncheol Choi

**Affiliations:** 1 Department of Anatomy, School of Medicine, Keimyung University, 1095 Dalgubeol-daero, Daegu 42601, Republic of Korea; 2 Department of Biomedical Laboratory Science, Dong-Eui Institute of Technology, 54 Yangji-ro, Busan 47230, Republic of Korea; 3 Department of Radiation Oncology, Keimyung University Dongsan Hospital, Keimyung University School of Medicine, Daegu 42601, Republic of Korea

**Keywords:** NKX3.2, liver hepatocellular carcinoma, TIMER, GEPIA2

## Abstract

*Background and Objectives*: The gene NKX3.2 plays a role in determining cell fate during development, and mutations of NKX3.2 have been studied in relation to human skeletal diseases. However, due to the lack of studies on the link between NKX3.2 and cancer, we aimed to provide insights into NKX3.2 as a new prognostic biomarker for liver hepatocellular carcinoma (LIHC). *Materials and Methods*: The clinical significance of LIHC was investigated using open gene expression databases. We comprehensively analyzed NKX3.2 expression in LIHC using Gene Expression Profiling Interactive Analysis 2, Tumor Immune Estimation Resource (TIMER), and Kaplan–Meier plotter databases. Then, we investigated the association between NKX3.2 expression and tumor-infiltrating immune cells (TIICs). *Results*: NKX3.2 expression was higher in the primary tumor group compared to the normal group, and expression was higher in fibrolamellar carcinoma (FLC) compared to other subtypes. When the prognostic value of NKX3.2 was evaluated, highly expressed NKX3.2 significantly improved the overall survival and had an unfavorable prognosis. In addition, NKX3.2 expression was associated with immune cell infiltration. Patients with low gene expression and high macrophage expression had a poorer survival rate than those with low NKX3.2 and low macrophage expression (*p* = 0.0309). *Conclusions*: High NKX3.2 expression may induce poorer prognosis in LIHC. In addition, these findings can be used as basic data due to the lack of available related research. However, further in vivo studies are essential to gain a deeper understanding of the biological role of NKX3.2 in LIHC and its potential implications for cancer development and progression.

## 1. Introduction

Liver hepatocellular carcinoma (LIHC) is the most common cancer worldwide with a poor prognosis [1,2]. The most common type of LIHC is hepatocellular carcinoma (HCC), which begins in the main type of liver cell, the hepatocyte. Its other types, such as intrahepatic cholangiocarcinoma and hepatoblastoma, are much less common. There are limited data on the treatment of advanced liver cancer, even though LIHC is the common cancer among solid tumors [3]. Moreover, advanced LIHC has high recurrence and metastasis rates, and a low survival rate [4]. To overcome this challenge, finding potential prognostic biomarkers through big data research is urgently needed. 

The first finding of NKX3.2 (Bapx1) was in Drosophila as a transcription factor [5,6]. It is a member of the NK family of developmental genes and has a central role in determining cell fate during the development of various organs and visceral mesoderm during midgut myogenesis [5]. NKX3.2 is involved in axial and limb skeleton formation and is known to protect chondrocytes from programmed apoptosis [7]; it is highly expressed in prochondrocytes and proliferating chondrocytes, but downregulated in mature chondrocytes, and plays a role in the hypertrophic maturation of articular chondrocytes in osteoarthritis [5,7,8]. NKX3.2 mutation is known as one of the genetic causes of spondylo-megaepiphyseal-metaphyseal dysplasia (SMMD), a human skeletal disease [9]. Interestingly, despite its roles in developmental and skeletal processes, research on NKX3.2 in the context of cancer has not been well-studied. Given its diverse functions and implications in developmental and skeletal processes, further exploration of NKX3.2′s potential involvement in cancer is warranted.

Herein, we demonstrated that NKX3.2 could be a target for the prediction of several cancers, for the first time. As a preliminary study on NKX3.2 in cancers, publicly available gene expression databases such as Total Cancer Genome Atlas (TCGA) and Tumor Immune Estimation Resource (TIMER) were used as recent studies [10,11]; NKX3.2 was highly expressed in most cancers compared to normal tissues. NKX3.2 expression was related to both cancer progression and rate of metastasis especially, in LIHC. Furthermore, we demonstrated that NKX3.2 expression was associated with the survival rate and immune cell infiltration in liver cancer patients. We conclude that NKX3.2 may be a novel biomarker for predicting prognosis and provides insight into the tumor immunogenicity of LIHC.

## 2. Materials and Methods

### 2.1. TIMER Database Analysis

TIMER is a TCGA database that includes immune infiltration and clinical data (age, sex, race, and stage) across 32 cancer types, for the *NKX3.2* gene. We analyzed *NKX3.2* gene expression and survival value, as well as the association between immune cell infiltration and cancer prognosis in LIHC, using correlation analysis among immune cells. In addition, the correlation between *NKX3.2* gene expression and TIICs, such as with CD4+ and CD8+ T cells, B cells, macrophages, neutrophils, and dendritic cells, was analyzed through the gene module, and gene expression levels were plotted against tumor purity. In addition, we analyzed the association between immune cell infiltration in LIHC. 

### 2.2. UALCAN Database Analysis

The UALCAN database (http://ualcan.path.uab.edu) uses TCGA level 3 RNA-seq and clinical data from LIHC (accessed on 1 February 2023). And then, the relative expression of genes across tumor and non-tumor tissues was compared according to cancer stages, tumor grade, or other clinicopathological features.

### 2.3. Immunohistochemistry (IHC) Staining Analysis

HPA (https://www.proteinatlas.org/) is a human proteome atlas database that contains information on protein distribution in human tissues and cells (accessed on 1 February 2023). To analyze the NKX3.2 proteomic expression levels, we obtained IHC images of NKX3.2 proteomic expression from the HPA for LIHC. The proteomic expression level of NKX3.2 was graded as not detected, low, medium, or high, based on the intensity of staining and the fraction of stained cells.

### 2.4. KM Database Analysis

KM is based on an online database, and includes survival rates, such as overall survival (OS), relapse-free survival (RFS), progression-free survival (PFS), disease-specific survival (DSS), and clinical data in patients with LIHC. Survival data of NKX3.2 were investigated using a log-rank test.

### 2.5. OSlihc Database Analysis

OSlihc (http://bioinfo.henu.edu.cn/DatabaseList.jsp) provides a platform for researchers to discover new prognostic biomarkers and may provide the opportunity to create novel targeted therapies for LIHC (accessed on 22 February 2023). To clarify the predictive value of genes in OSlihc, survival data such as OS, disease-free interval (DFI), progression-free interval (PFI), and DSS were generated, and OS was measured in all cohorts and combined cohorts, while DFI, PFI, and DSS were analyzed using TCGA.

### 2.6. LinkedOmics Database Analysis 

The LinkedOmics database (http://www.linkedomics.org/admin.php) is a web-based platform for analyzing 32 TCGA cancer-associated multidimensional datasets (accessed on 26 February 2023). Correlation in NKX3.2 associated genes was analyzed by Pearson’s correlation coefficient, and presented in volcano plots, heat maps, or scatter plots. 

Gene Ontology (GO) and Kyoto Encyclopedia of Genes and Genomes (KEGG) pathway enrichment analyses were conducted using the Database for Annotation, Visualization, and Integrated Discovery (DAVID 6.8, v6.8; https://david.ncifcrf.gov/home.jsp) and the bioinformatics online tool (http://www.bioinformatics.com.cn) (accessed on 4 March 2023).

### 2.7. GEPIA2 Database Analysis

GEPIA2 (http://gepia2.cancer-pku.cn/#index) was used to integrate the clinical data acquired from 9736 tumors and 8587 normal tissues from TCGA and GTEx projects (accessed on 9 March 2023). GEPIA2 performs survival analyses according to gene or isoform expression levels. Based on gene expression in 33 different types of cancer, OS and RFS were generated by GEPIA2. Heatmaps of OS and DFS based on NKX3.2 gene expression across all TCGA tumor types were obtained in the “Survival Map” module. GEPIA2 showed KM curves based on NKX3.2 gene expression with the log-rank test and the Mantel–Cox test.

### 2.8. Statistical Analysis

Gene expression data were analyzed using online tools and the TIMER database. Survival analyses were performed by KM plots, TIMER, and OSlihc. Survival results are presented as HR and *p* values by a log-rank test. The correlation between gene expression and immune signature score was performed by Spearman’s correlation. All data in this study were extracted from public open databases, and we conducted statistical analysis by web tools. All results are presented as *p*-values from the log-rank test. Statistical significance was set at *p* < 0.05.

## 3. Results

### 3.1. Assessment of NKX3.2 Expression in Different Cancer and Normal Tissues

**Figure 1:**
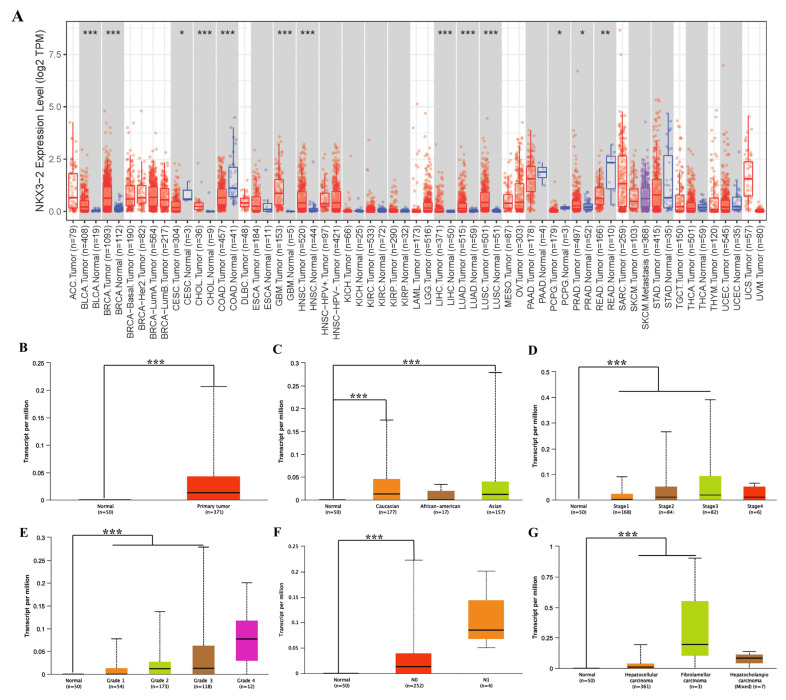
Clinical characteristics according to *NKX3.2* gene expression. (**A**) Expression levels of normal and tumor tissues of NKX3.2 in various types of cancer. (**B**) Transcription level of NKX3.2 between LIHC tissue and normal tissue. (**C**) Relationship with race in LIHC according to NKX3.2 expression. (**D**,**E**) Association with LIHC stage. (**F**,**G**) Association with nodular metastasis according to NKX3.2 and histological subtypes in LIHC. * *p* < 0.05, ** *p* < 0.01 and *** *p* < 0.001.

TIMER was used to examine RNA sequencing data from TCGA to evaluate how NKX3.2 expression differs in specific tumor types. NKX3.2 expression between normal and tumor tissues significantly differs in breast invasive carcinoma (*p* < 0.05), bladder urothelial carcinoma (*p* < 0.001), cholangiocarcinoma (*p* < 0.001), glioblastoma multiforme (*p* < 0.001), head and neck squamous carcinoma (*p* < 0.001), lung squamous cell carcinoma (*p* < 0.001), lung adenocarcinoma (*p* < 0.001), rectal cancer (*p* < 0.05), and LIHC (*p* < 0.001) (Figure 1A). The results revealed that NKX3.2 was expressed highly in most cancers. 

We specifically investigated the clinical characteristics of NKX3.2 expression in LIHC using the UALCAN database to validate the results from TIMER. The *NKX3.2* gene showed significant results in Asian and Caucasian patients. In addition, it was overexpressed in the primary tumor group compared to the normal group (Figure 1B), and its expression level was significantly different according race (Figure 1C). NKX3.2 was more significantly expressed with increasing tumor histological and clinical stages (Figure 1D,E). Furthermore, lymph node metastasis was associated with NKX3.2 expression (Figure 1F). When compared to other histological subtypes, NKX3.2 showed higher expression in FLC (Figure 1G).

### 3.2. Protein Expression of NKX3.2 in LICH 

**Figure 2:**
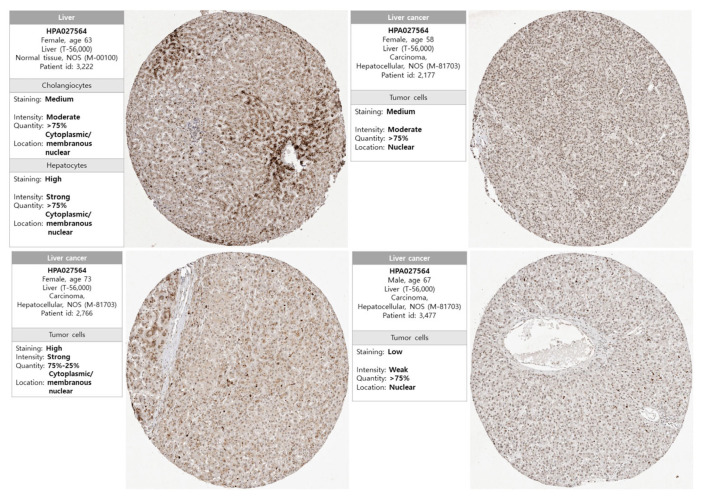
Protein expression levels of NKX3.2 in LIHC. Protein expression of NKX3.2 was analyzed using the Human Protein Atlas (HPA) database.

To assess the expression of NKX3.2 at the protein level, the protein expression levels of NKX3.2 were obtained from the HPA database. IHC images from the HPA revealed that the protein expression of NKX3.2 was undetected in normal liver tissue, whereas protein expression of NKX3.2 was significantly higher in LICH tissues than in normal tissues (Figure 2). Our results revealed overexpression of NKX3.2 at transcriptional and translational levels in patients with LIHC.

### 3.3. Multifaceted Prognostic Value of NKX3.2 in LIHC

The current study used three databases to evaluate NKX3.2 in LIHC. To date, the prognostic value of NKX3.2 has not been reported, and the correlation between the expression level of NKX3.2 and prognosis in LIHC were evaluated using the GEPIA2 database. These results demonstrated the effect of the expression level of NKX3.2 on cancer survival and recurrence rates. 

**Figure 3:**
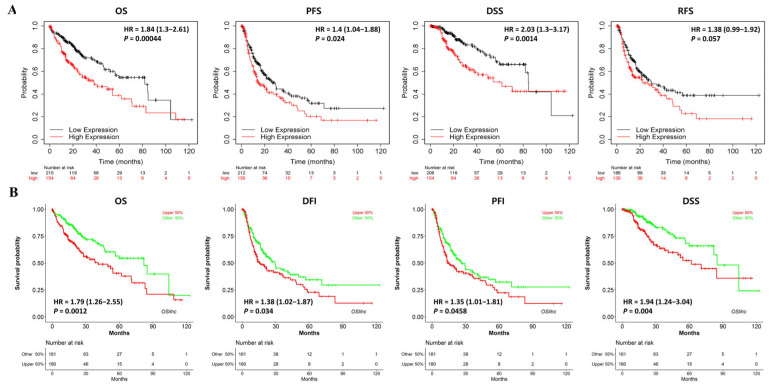
Prognostic significance of high expression of NKX3.2 in LIHC. (**A**) The prognostic value of NKX3.2 was analyzed using KM. (**B**) Evaluation of the prognostic value of NKX3.2 in Oslihc. *p*-value, confidence interval (95%CI) and number at risk are as shown. The y-axis represents survival rate and the x-axis represents survival time (months).

The prognostic significance of NKX3.2 expression was analyzed in LIHC using the Kaplan–Meier method. NKX3.2 with high expression showed poor overall survival (OS) (HR  =  1.84, *p*  < 0.001), progression-free survival (PFS; HR  =  1.4, *p*  =  0.024), and disease-specific survival (DSS; HR = 2.03, *p* = 0.0014). Similarly, recurrence-free survival (RFS) was also related to patients with high expression of NKX3.2 (HR = 1.38, *p* = 0.057); however, this was not statistically significant (Figure 3A).

We used the OSlihc database to identify NKX3.2 as a new biomarker candidate and confirmed a significant association with survival. Patients with a high expression of NKX3.2 in LIHC had OS (HR: 1.7913, *p* = 0.0012), DFS (HR: 1.3837, *p* = 0.034), PFS (HR = 1.3507, *p* = 0.0458), and DSS (HR = 1.9388, *p* = 0.004), which indicated a poor prognosis. The increased expression of NKX3.2 in LIHC indicated an adverse prognosis (Figure 3B). Its prognostic value, stratified using clinical characteristics, is summarized in Figure S1.

### 3.4. Association of NKX3.2 Expression with Immune Cell Infiltration in LIHC

**Figure 4:**
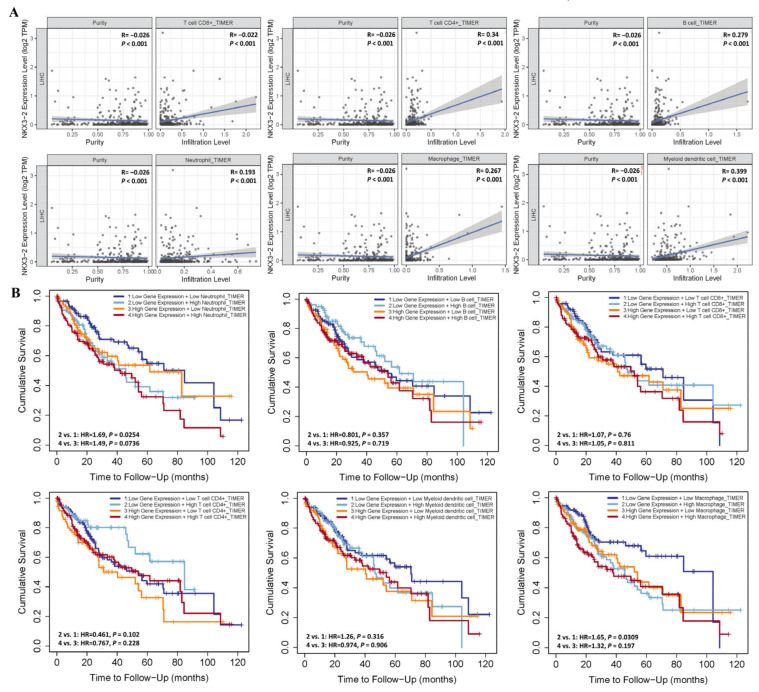
Correlation between NKX3.2 expression and infiltrating immune cells in LIHC. (**A**) The correlation between NKX3.2 and infiltrating immune cells was analyzed using the TIMER database. (**B**) Survival analysis according to NKX3.2 gene expression and expression of TIICs.

Immune cell infiltration usually accelerates cancer progression and affects survival outcomes. Therefore, we examined the association between NKX3.2 and six tumor immune-infiltrating cells using the TIMER database. Neutrophils, macrophages, and CD8+ T, CD4+ T, B, and myeloid dendritic cells all showed a positive correlation. The results were NKX3.2 and CD8+ T cells (rho = −0.022, *p* < 0.001), CD4+ T cells (rho = 0.34, *p* = < 0.001), and B cells (rho = 0.279, *p* < 0.001), macrophages (rho = 0.267, *p* < 0.001), neutrophils (rho = 0.193, *p* < 0.001) and dendritic cells (rho = 0.399, *p* < 0.001) (Figure 4A). 

We then investigated the correlation between the tumor immune infiltrating cells, NKX3.2 expression, and survival time in LIHC. The results showed that patients with high NKX3.2 and high neutrophil expressions had a lower survival rate than patients with high gene expression and low neutrophil expression, though it did not achieve statistical significance (*p* = 0.0763). The patient group with low gene expression and high macrophage expression had a poor survival rate compared to the patient group with low NKX3.2 and low macrophage expression (*p* = 0.0309) 

Also, we investigated whether high NKX3.2 expression was related with prognosis and TIICs in LIHC. Patients with high NKX3.2 expression and high neutrophil infiltration levels showed worse prognosis than those with low NKX3.2 expression and low neutrophil infiltration levels. Low NKX3.2 expression and high neutrophil infiltration levels were associated with a worse prognosis than low NKX3.2 expression and low neutrophil infiltration levels. High NKX3.2 expression and low CD4+T cell infiltration levels were associated with a worse prognosis than low NKX3.2 expression and high CD4+T cell infiltration levels. High NKX3.2 expression and high macrophage infiltration levels predicted a worse result than low NKX3.2 expression and low macrophage infiltration levels. Moreover, low NKX3.2 expression and high macrophage infiltration levels were associated with a worse prognosis than low NKX3.2 expression and low macrophage infiltration levels. B cells, CD8+T cells, and dendritic cells showed no significant level of prognosis (Figure 4B).

Therefore, the analysis of the cumulative curve indicated that the infiltration of immune cells was associated with the NKX3.2 gene in LIHC, and affected the prognosis. These data suggest that NKX3.2 can be the prediction gene for the immune invasion process in LIHC.

### 3.5. Co-Expression Analysis of the NKX3.2 Gene

**Figure 5:**
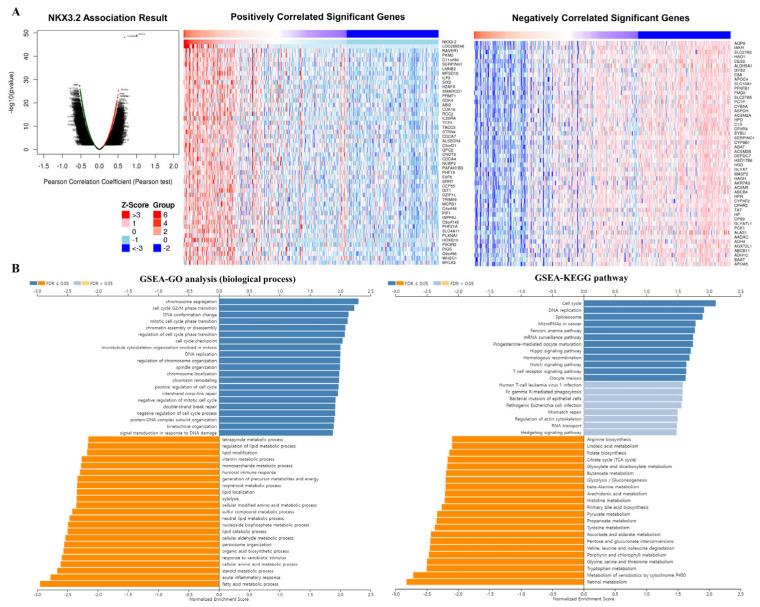
Correlation of NKX3.2 and related genes in LIHC. (**A**) The volcanic map showed correlations between NKX3.2 and genes differentially expressed in LIHC. Top 50 genes positively associated with NKX3.2 in LIHC and top 50 genes negatively associated with NKX3.2 in LIHC (**B**) GO and KEGG analyses performin LIHC.

To obtain biological insights into NKX3.2 in LIHC, we analyzed the co-expressed genes of NKX3.2 using the LinkedOmics database, and aimed to identify differentially expressed genes that correlated with the *NKX3.2* gene in LIHC. By Pearson correlation analysis, we determined positively and negatively correlated differentially expressed genes for NKX3.2. Of the 19,914 genes analyzed, 13,131 were positively correlated with the *NKX3.2* gene in LIHC, and 6782 were negatively correlated. Volcano plots showing these correlations are depicted in Figure 5A (Left). Additionally, we picked 50 genes to make lists of positive and negative correlations with the *NKX3.2* gene based on our analysis (Figure 5A (Right)). The top five positively correlated genes were NKX3.2, LOC285548, RAVER1, PKM2, and C11orf84, and the top five negatively correlated genes were aQP9, tAKR, SLC27A2, HAO1, and CES2. Furthermore, GO (biological process, cellular component, and molecular function) and KEGG pathway analyses of NKX3.2-related genes were performed. GO annotations presented that NKX3.2 co-expressed genes were predominantly co-expressed during cell cycle G2/M phase transition, chromosome segregation, DNA conformation and change, mitotic cell cycle phase transition, cell cycle checkpoint, etc. (Figure 5B, Left). KEGG pathway assays demonstrated enrichment of the cell cycle, DNA replication, spliceosome, and microRNAs in cancer (Figure 5B, Right).

### 3.6. Prognosis Analysis of the NKX3.2-Related Genes

**Figure 6:**
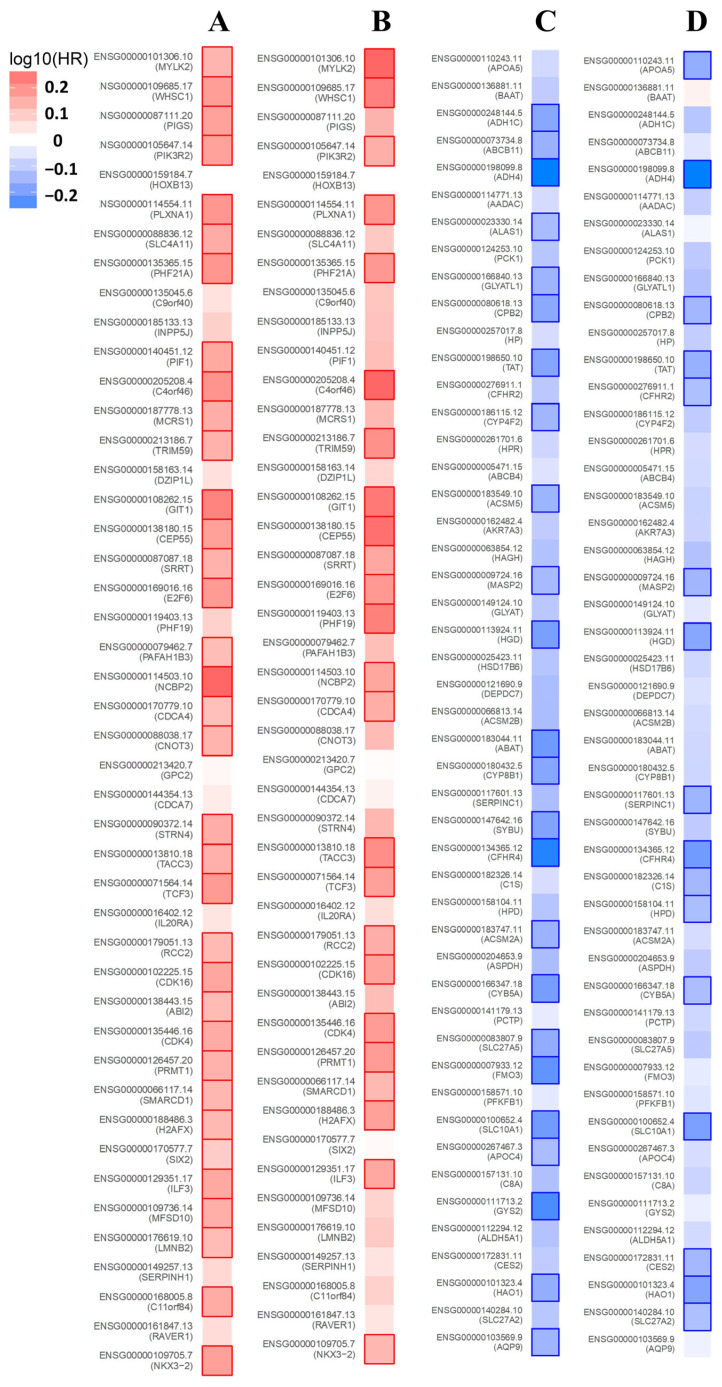
Prognostic value of NKX3.2-related genes in LIHC. Survival map of the positively related genes of NKX3.2 in OS (**A**) and RFS (**B**). Survival map of the negatively related genes of NKX3.2 in OS (**C**) and RFS (**D**). Survival map presenting the log10 (HR) of the genes in LIHC. A square with bold border represents a *p* value < 0.05 in the survival analysis.

We investigated the prognostic value of NKX3.2-related genes in LIHC using data from the ZEPIA2 database. NKX3.2-related genes are most likely to be high-risk genes for LIHC. Among the positively related genes of NKX3.2, 35 showed a high hazard ratio (HR) for OS (Figure 6A), and 24 showed a high HR for RFS (Figure 6B). In contrast, 24 genes of the negative genes had low HR (*p* < 0.05) in OS (Figure 6C), and 16 genes had low HR in RFS (Figure 6D). Also, we showed that positively related genes of NKX3.2 indicated a high HR in different types of cancer, and the negative genes indicated a low HR in different types of cancer (Supplementary Figure S1). Therefore, NKX3.2-related genes have prognostic significance in various types of cancer, including LIHC.

## 4. Discussion

LIHC is a malignant tumor, and its incidence is continuously increasing. After primary treatment, such as surgical resection and transplantation, LIHC has a high recurrence rate and 5-year survival rate of 50–70% [12]. To date, NKX3.2 has been studied extensively; however, our findings are the first to show the relation with cancer. This study will help to predict the prognosis of patients with LIHC and improve future outcomes. 

*NKX3.2*, a well-known gene for its roles in developmental processes and human skeletal diseases, has not been previously studied in the context of cancer. Through comprehensive analyses of gene expression databases, we have identified a significant correlation between NKX3.2 expression and clinical characteristics, especially in LIHC.

The pathological types in which LIHC develops occur in chronic hepatitis and fibrosis [13]. In particular, viral hepatitis infection is a potential risk factor for LIHC. Asian and Caucasian patients showed a higher expression of NKX3.2 in LIHC; however, only Asian patients had significant OS results. According to previous studies, 60% of Asian patients with LIHC are HBV-positive, whereas only 25% of European patients are carriers [14,15]. Similarly, OS results from men showed poor outcomes compared to results from women. NKX3.2 will be a significant target to predict other types of liver diseases, such as chronic hepatitis, cirrhosis, and non-alcoholic fatty liver disease. Therefore, NKX3.2 may have an important role in the carcinogenesis of Asian patients with LIHC. 

Primary liver cancer is a frequent cancer worldwide, and hepatocellular carcinoma (HCC) is the main type of primary liver cancer, accounting for 75–85% of diagnoses [16]. Our results show that NKX3.2 expression is high in primary tumors. Additionally, FLC, as a rare and unknown cancer type, is more prevalent in younger patients than other subtypes [17,18]. Recent studies have shown that FLC has a better overall prognosis than other primary liver tumors such as HCC and intrahepatic cholangiocarcinoma. However, due to the lack of data on FLC, it is said that it is difficult to develop new treatments. Also, because of the rarity of FLC, it has an aggressive tumor with a low overall cure rate, and recurrence is common even after surgery [18]. In this study, NKX3.2 was overexpressed in FLC. Our study is limited because it is unknown whether a high expression of NKX3.2 causes primary tumors and FLC. Additional experiments will be helpful in the development of therapeutic agents.

Regarding immunotherapy, the tumor microenvironment (TME) is known to affect the efficacy of immunotherapy [19,20]. As far as is known, studies have shown that immunotherapy provides a survival advantage in LIHC treatment [21,22]. In this study, the level of immune infiltration of NKX3.2 in LIHC was explored. LIHC is an inflammation-related cancer and is known to be closely related to the immune response [23]. In particular, the immune response of LIHC is regulated by various activating and inhibitory signaling pathways. Notably, NKX3.2 plays an important role in regulating LIHC immunology. Immunopenetration analysis showed that NKX3.2 was significantly associated with various immune cells in LIHC. Analyses using the TIMER database showed that NKX3.2 was positively correlated with the infiltration status of immune cells including neutrophils, macrophages, and CD4+ T, CD8+ T, B, and dendritic cells. Therefore, TIICs are closely related to tumorigenesis and its development. In our study, high NKX3.2 expression and cell infiltration of neutrophils showed a worse prognosis than low NKX3.2 expression. 

According to previous studies, inflammation is essential for cancer initiation by damaging tissues, and neutrophils play an essential role in this process [24]. In addition, neutrophils have recently received a lot of attention because they are associated with cancer growth promotion and metastasis [24]. Future studies are needed to determine the role of the NKX3.2 gene in neutrophil expression.

It is also important to note that although some associations between NKX3.2 expression, immune cell infiltration and survival outcomes were observed, not all reached statistical significance. Further studies and studies with larger sample sizes are needed to validate these results and understand the precise mechanism for the role of NKX3.2 in immune invasion and cancer progression in LIHC.

GO and KEGG enrichment analyses of the cell cycle, DNA replication, translation initiation and chromosome segregation, and G2/M phase transition indicate that NKX3.2 is involved in cell proliferation. Especially, the KEGG pathway analysis demonstrated enrichment in pathways related to the cell cycle, DNA replication, and spliceosome. These pathways are vital for fundamental cellular processes, including cell division, DNA synthesis, and RNA processing. The enrichment of these pathways further supports the potential role of NKX3.2 in tumor growth and progression in LIHC. These results indicate that NKX3.2 may contribute to LIHC development through DNA replication and other physiological processes. The specific mechanism of NKX3.2 in LIHC has not been reported in detail and requires further research. the analysis of co-expressed genes and functional annotations provided valuable biological insights into the potential role of NKX3.2 in LIHC. These findings suggest that NKX3.2 may play a significant role in cell cycle regulation and DNA-related processes in LIHC, and its dysregulation could be involved in the development and progression of liver cancer. However, further experimental studies are needed to validate these findings and uncover the specific mechanisms through which NKX3.2 contributes to LIHC pathogenesis.

## 5. Conclusions

In conclusion, our findings show that NKX3.2 can be a new prediction marker for LIHC based on our multiple analyses with immune cell infiltration and survival rate, for the first time. Due to the lack of bio-markers for liver diseases, it is hard to develop and find new drugs. Combining the mechanism of NKX3.2 with our predictions is the key to developing proper therapeutic strategies in LIHC and other types of liver diseases. 

## Data Availability

The data presented in this study are available on request from the corresponding author.

## Supplementary Materials

The following supporting information can be downloaded at: https://www.mdpi.com/article/10.3390/medicina59101782/s1, Figure S1: Prognostic value of NKX3.2-related genes in various cancers. Survival map of the positive-related genes of NKX3.2 in OS and RFS (A). Survival map of the negative-related genes of NKX3.2 in OS and RFS (B). Survival map presenting the log10 (HR) of the genes in LIHC. A square with bold border represents a *p* value < 0.05 in the survival analysis.

## References

[1] Wang Z., Zhang G., Wu J., Jia M. (2013). Adjuvant therapy for hepatocellular carcinoma: Current situation and prospect. Drug Discov. Ther..

[2] Alqahtani A., Khan Z., Alloghbi A., Ahmed T.S.S., Ashraf M., Hammouda D.M. (2019). Hepatocellular Carcinoma: Molecular Mechanisms and Targeted Therapies. Medicina.

[3] Mulero-Sánchez A., Ramirez C.F.A., du Chatinier A., Wang H., Koomen S.J.I., Song J., de Groot M.H.P., Lieftink C., Bosma A., Burylo A. (2023). Rational combination of SHP2 and mTOR inhibition for the treatment of hepatocellular carcinoma. Mol. Oncol..

[4] Fang X., Yan Q., Liu S., Guan X.Y. (2022). Cancer Stem Cells in Hepatocellular Carcinoma: Intrinsic and Extrinsic Molecular Mechanisms in Stemness Regulation. Int. J. Mol. Sci..

[5] Waldmann L., Leyhr J., Zhang H., Öhman-Mägi C., Allalou A., Haitina T. (2021). The broad role of Nkx3.2 in the development of the zebrafish axial skeleton. PLoS ONE.

[6] Lettice L., Hecksher-Sørensen J., Hill R. (2001). The role of Bapx1 (Nkx3.2) in the development and evolution of the axial skeleton. J. Anat..

[7] Kim J.A., Im S., Cantley L.C., Kim D.W. (2015). Suppression of Nkx3.2 by phosphatidylinositol-3-kinase signaling regulates cartilage development by modulating chondrocyte hypertrophy. Cell. Signal..

[8] Rainbow R.S., Won H.K., Zeng L. (2014). The role of Nkx3.2 in chondrogenesis. Front. Biol..

[9] Simon M., Campos-Xavier A.B., Mittaz-Crettol L., Valadares E.R., Carvalho D., Speck-Martins C.E., Nampoothiri S., Alanay Y., Mihci E., van Bever Y. (2012). Severe neurologic manifestations from cervical spine instability in spondylo-megaepiphyseal-metaphyseal dysplasia. Am. J. Med. Genet. Part C Semin. Med. Genet..

[10] Kim J., Park J.H., Lee J.H. (2021). Analysis of the Cancer Genome Atlas Data to Determine the Prognostic Value of GABPB1L and TERT in Glioblastoma. Keimyung Med. J..

[11] Kim J., Jung S.J., Lee J.H. (2022). Clinical and Prognostic Values of DNMT3B Expression in Hepatocellular Carcinoma. Keimyung Med. J..

[12] Gil Lee J., Kang C.M., Park J.S., Kim K.S., Yoon D.S., Choi J.S., Lee W.J., Kim B.R. (2006). The actual five-year survival rate of hepatocellular carcinoma patients after curative resection. Yonsei Med. J..

[13] Matsumura H., Nirei K., Nakamura H., Higuchi T., Arakawa Y., Ogawa M., Tanaka N., Moriyama M. (2013). Histopathology of type C liver disease for determining hepatocellular carcinoma risk factors. World J. Gastroenterol..

[14] Pollack H.J., Kwon S.C., Wang S.H., Wyatt L.C., Trinh-Shevrin C., AAHBP Coalition (2014). Chronic hepatitis B and liver cancer risks among Asian immigrants in New York City: Results from a large, community-based screening, evaluation, and treatment program. Cancer Epidemiol. Biomark. Prev..

[15] Rizzo G.E.M., Cabibbo G., Craxì A. (2022). Hepatitis B Virus-Associated Hepatocellular Carcinoma. Viruses.

[16] Llovet J.M., Kelley R.K., Villanueva A., Singal A.G., Pikarsky E., Roayaie S., Lencioni R., Koike K., Zucman-Rossi J., Finn R.S. (2021). Hepatocellular carcinoma. Nat. Rev. Dis. Primers.

[17] Lalazar G., Simon S.M. (2018). Fibrolamellar Carcinoma: Recent Advances and Unresolved Questions on the Molecular Mechanisms. Semin. Liver Dis..

[18] Alshareefy Y., Shen C.Y., Prekash R.J. (2023). Exploring the molecular pathogenesis, diagnosis and treatment of fibrolamellar hepatocellular carcinoma: A state of art review of the current literature. Pathol. Res. Pract..

[19] Son B., Lee S., Youn H., Kim E., Kim W., Youn B. (2017). The role of tumor microenvironment in therapeutic resistance. Oncotarget.

[20] Cao R., Yuan L., Ma B., Wang G., Tian Y. (2021). Tumour microenvironment (TME) characterization identified prognosis and immunotherapy response in muscle-invasive bladder cancer (MIBC). Cancer Immunol. Immunother..

[21] Xu F., Jin T., Zhu Y., Dai C. (2018). Immune checkpoint therapy in liver cancer. J. Exp. Clin. Cancer Res..

[22] Zhu X.D., Tang Z.Y., Sun H.C. (2020). Targeting angiogenesis for liver cancer: Past, present, and future. Genes Dis..

[23] Le Y., Kong H., Gao X., Zhu J. (2022). Prognostic and Immunological Significance of FUNDC1 in Hepatocellular Carcinoma: A Study on TCGA Mining. Comput. Math. Methods Med..

[24] Xiong S., Dong L., Cheng L. (2021). Neutrophils in cancer carcinogenesis and metastasis. J. Hematol. Oncol..

