# Supplementary material for: Comprehensive Analysis of NKX3.2 in Liver Hepatocellular Carcinoma by Bigdata
Source: Medicina (Kaunas). 2023 Oct 6;59(10):1782. doi: 10.3390/medicina59101782 (PMC10608539; doi:10.3390/medicina59101782)
Supplement: Supplementary file 1 [file medicina-59-01782-s001.zip › medicina-2615891-supplementary.pdf]

(A)

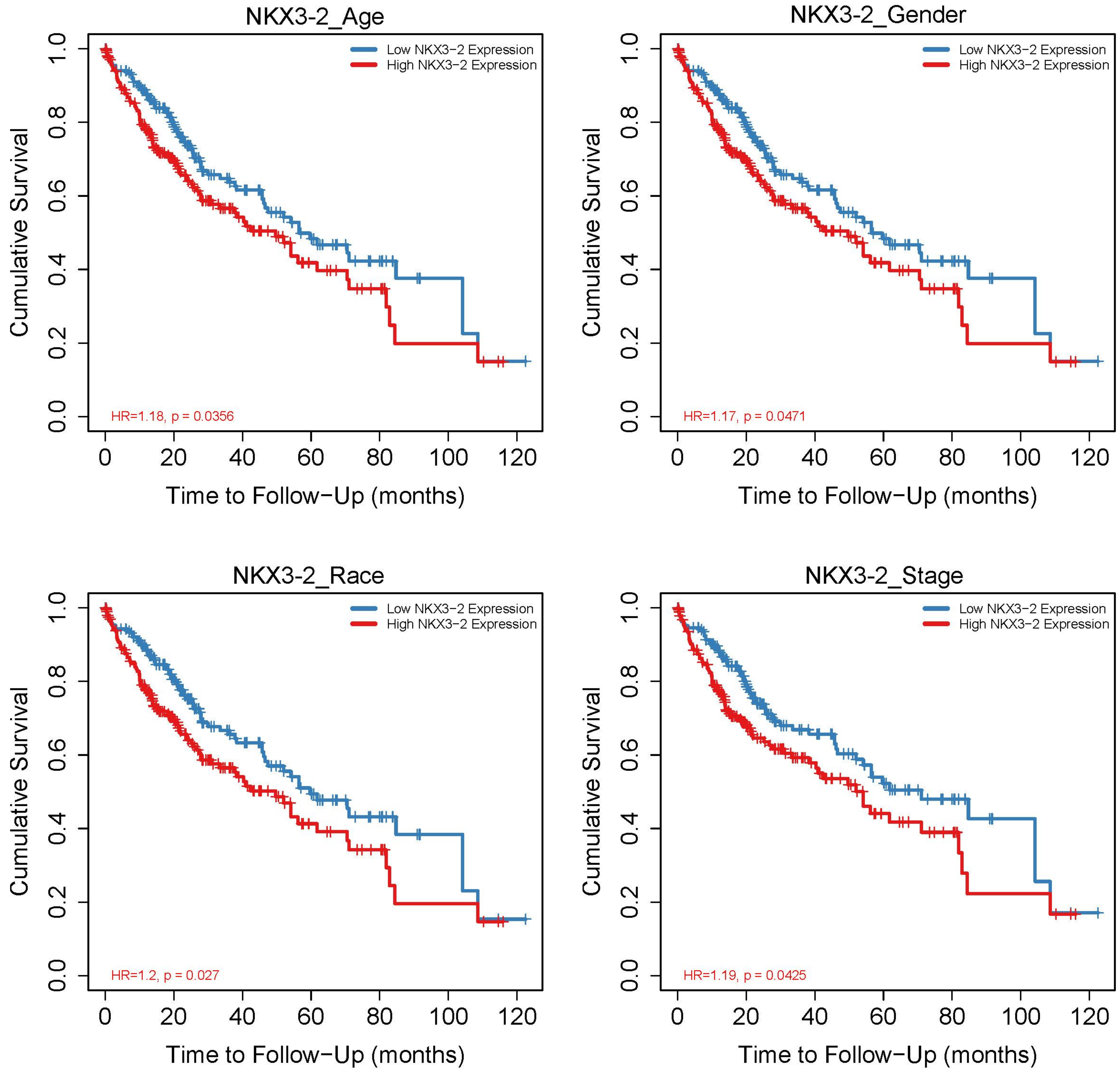

Figure S1. Clinical characteristics according to NKX3.2 gene expression. (A) Clinical characteristics analyzed by the KM database. (B-E) Prognostic value according to the expression of NKX3.2 in LIHC using the osilic database.

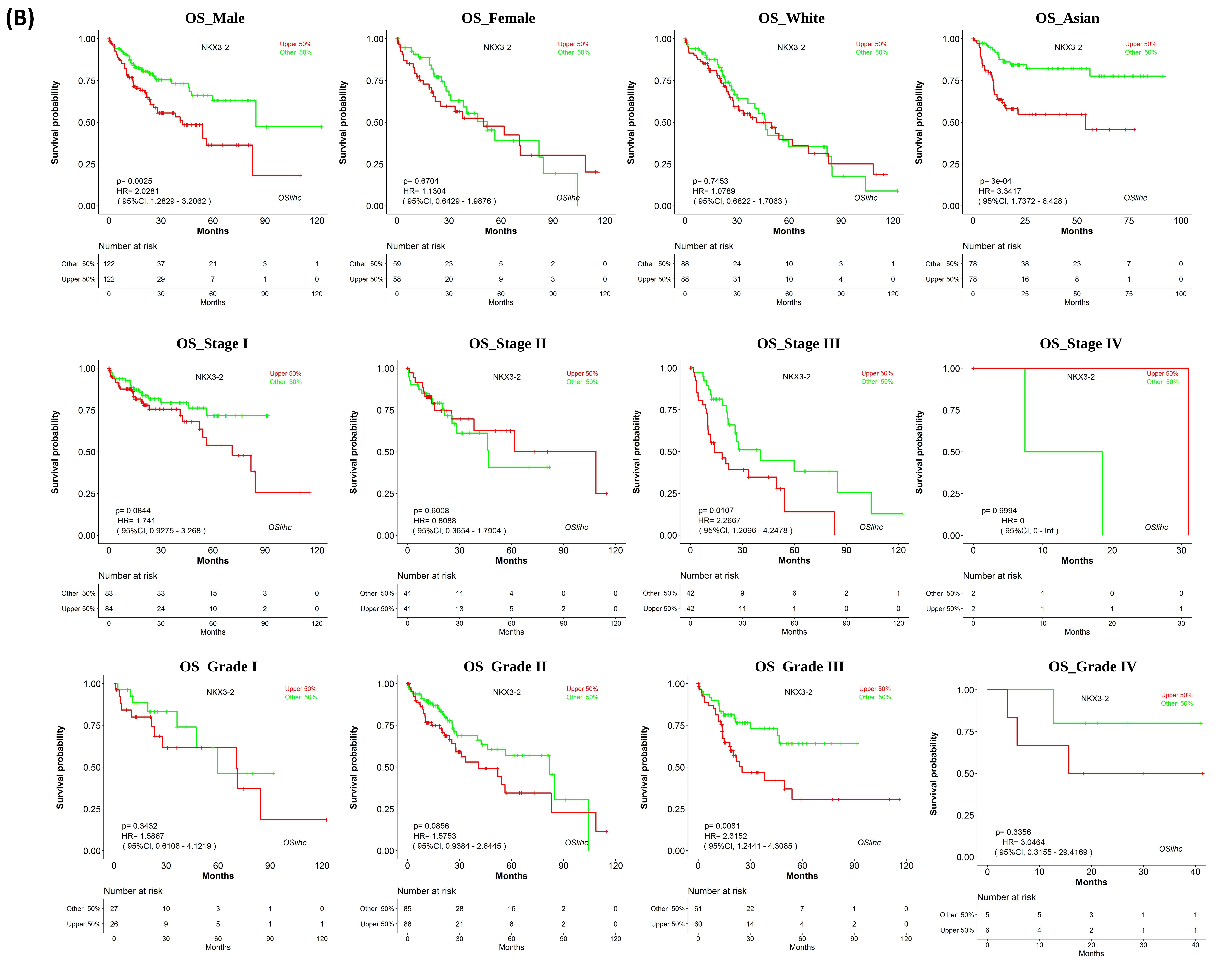
(B)
OS_Male
NKX3-2
Upper 50%
Other 50%
p= 0.0025
HR= 2.0281
(95%CI, 1.2829 - 3.2062)
OSlihc
Survival probability
Months
Number at risk
Other 50% 122 37 21 3 1
Upper 50% 122 29 7 1 0
OS_Female
p= 0.6704
HR= 1.1304
(95%CI, 0.6429 - 1.9876)
Other 50% 59 23 5 2 0
Upper 50% 58 20 9 3 0
OS_White
p= 0.7453
HR= 1.0789
(95%CI, 0.6822 - 1.7063)
Other 50% 88 24 10 3 1
Upper 50% 88 31 10 4 0
OS_Asian
p= 3e-04
HR= 3.3417
(95%CI, 1.7372 - 6.428)
Other 50% 78 38 23 7 0
Upper 50% 78 16 8 1 0
OS_Stage I
p= 0.0844
HR= 1.741
(95%CI, 0.9275 - 3.268)
Other 50% 83 33 15 3 0
Upper 50% 84 24 10 2 0
OS_Stage II
p= 0.6008
HR= 0.8088
(95%CI, 0.3654 - 1.7904)
Other 50% 41 11 4 0 0
Upper 50% 41 13 5 2 0
OS_Stage III
p= 0.0107
HR= 2.2667
(95%CI, 1.2096 - 4.2478)
Other 50% 42 9 6 2 1
Upper 50% 42 11 1 0 0
OS_Stage IV
p= 0.9994
HR= 0
(95%CI, 0 - Inf)
Other 50% 2 1 0 0
Upper 50% 2 1 1 1
OS_Grade I
p= 0.3432
HR= 1.5867
(95%CI, 0.6108 - 4.1219)
Other 50% 27 10 3 1 0
Upper 50% 26 9 5 1 1
OS_Grade II
p= 0.0856
HR= 1.5753
(95%CI, 0.9384 - 2.6445)
Other 50% 85 28 16 2 0
Upper 50% 86 21 6 2 0
OS_Grade III
p= 0.0081
HR= 2.3152
(95%CI, 1.2441 - 4.3085)
Other 50% 61 22 7 1 0
Upper 50% 60 14 4 2 0
OS_Grade IV
p= 0.3356
HR= 3.0464
(95%CI, 0.3155 - 29.4169)
Other 50% 5 5 3 1 1
Upper 50% 6 4 2 1 1

(C)

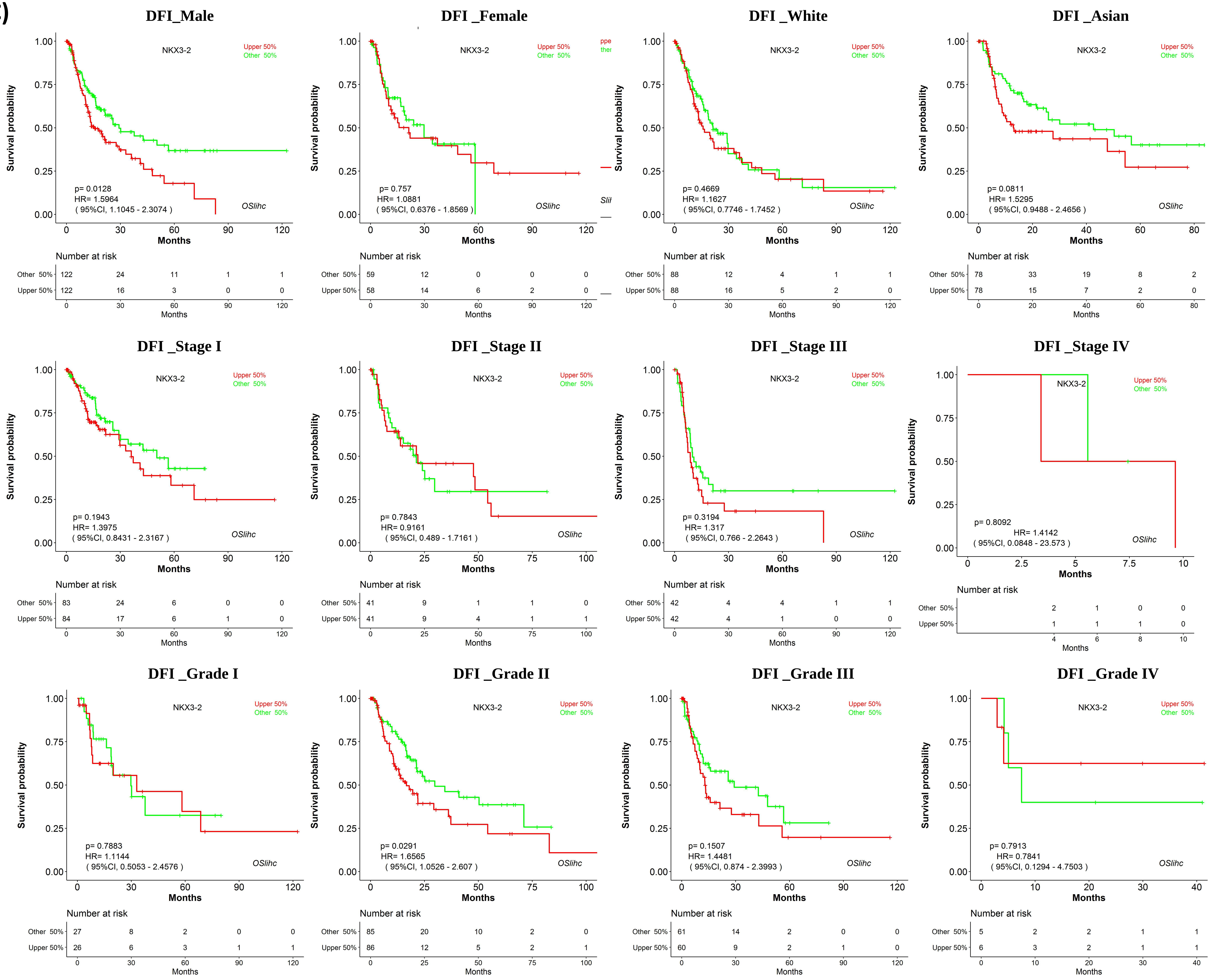
DFI_Male
NKX3-2
Upper 50%
Other 50%
p= 0.0128
HR= 1.5964
(95%CI, 1.1045 - 2.3074)
OSlihc
DFI _Female
NKX3-2
Upper 50%
Other 50%
p= 0.757
HR= 1.0881
(95%CI, 0.6376 - 1.8569)
OSlihc
DFI _White
NKX3-2
Upper 50%
Other 50%
p= 0.4669
HR= 1.1627
(95%CI, 0.7746 - 1.7452)
OSlihc
DFI _Asian
NKX3-2
Upper 50%
Other 50%
p= 0.0811
HR= 1.5295
(95%CI, 0.9488 - 2.4656)
OSlihc
DFI _Stage I
NKX3-2
p= 0.1943
HR= 1.3975
(95%CI, 0.8431 - 2.3167)
OSlihc
DFI _Stage II
NKX3-2
p= 0.7843
HR= 0.9161
(95%CI, 0.489 - 1.7161)
OSlihc
DFI _Stage III
NKX3-2
p= 0.3194
HR= 1.317
(95%CI, 0.766 - 2.2643)
OSlihc
DFI _Stage IV
NKX3-2
p= 0.8092
HR= 1.4142
(95%CI, 0.0848 - 23.573)
OSlihc
DFI _Grade I
NKX3-2
p= 0.7883
HR= 1.1144
(95%CI, 0.5053 - 2.4576)
OSlihc
DFI _Grade II
NKX3-2
p= 0.0291
HR= 1.6565
(95%CI, 1.0526 - 2.607)
OSlihc
DFI _Grade III
NKX3-2
p= 0.1507
HR= 1.4481
(95%CI, 0.874 - 2.3993)
OSlihc
DFI _Grade IV
NKX3-2
p= 0.7913
HR= 0.7841
(95%CI, 0.1294 - 4.7503)
OSlihc
Survival probability
Months
Number at risk

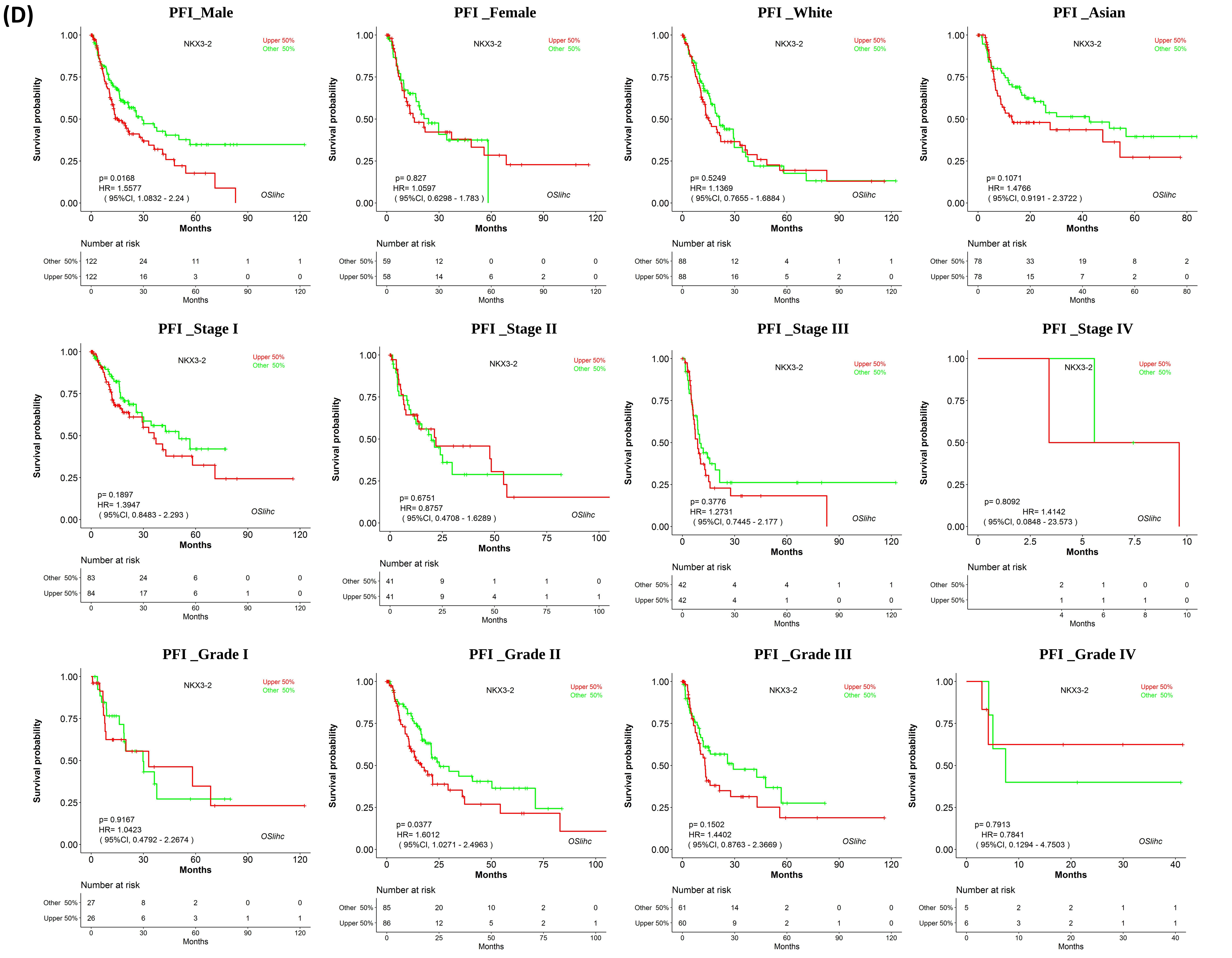
(D)
PFI_Male
NKX3-2
Upper 50%
Other 50%
p= 0.0168
HR= 1.5577
(95%CI, 1.0832 - 2.24)
OSlihc
Number at risk
Other 50% 122 24 11 1 1
Upper 50% 122 16 3 0 0
PFI _Female
p= 0.827
HR= 1.0597
(95%CI, 0.6298 - 1.783)
Other 50% 59 12 0 0 0
Upper 50% 58 14 6 2 0
PFI _White
p= 0.5249
HR= 1.1369
(95%CI, 0.7655 - 1.6884)
Other 50% 88 12 4 1 1
Upper 50% 88 16 5 2 0
PFI _Asian
p= 0.1071
HR= 1.4766
(95%CI, 0.9191 - 2.3722)
Other 50% 78 33 19 8 2
Upper 50% 78 15 7 2 0
PFI _Stage I
p= 0.1897
HR= 1.3947
(95%CI, 0.8483 - 2.293)
Other 50% 83 24 6 0 0
Upper 50% 84 17 6 1 0
PFI _Stage II
p= 0.6751
HR= 0.8757
(95%CI, 0.4708 - 1.6289)
Other 50% 41 9 1 1 0
Upper 50% 41 9 4 1 1
PFI _Stage III
p= 0.3776
HR= 1.2731
(95%CI, 0.7445 - 2.177)
Other 50% 42 4 4 1 1
Upper 50% 42 4 1 0 0
PFI _Stage IV
p= 0.8092
HR= 1.4142
(95%CI, 0.0848 - 23.573)
Other 50% 2 1 0 0
Upper 50% 1 1 1 0
PFI _Grade I
p= 0.9167
HR= 1.0423
(95%CI, 0.4792 - 2.2674)
Other 50% 27 8 2 0 0
Upper 50% 26 6 3 1 1
PFI _Grade II
p= 0.0377
HR= 1.6012
(95%CI, 1.0271 - 2.4963)
Other 50% 85 20 10 2 0
Upper 50% 86 12 5 2 1
PFI _Grade III
p= 0.1502
HR= 1.4402
(95%CI, 0.8763 - 2.3669)
Other 50% 61 14 2 0 0
Upper 50% 60 9 2 1 0
PFI _Grade IV
p= 0.7913
HR= 0.7841
(95%CI, 0.1294 - 4.7503)
Other 50% 5 2 2 1 1
Upper 50% 6 3 2 1 1
Survival probability
Months

(E)

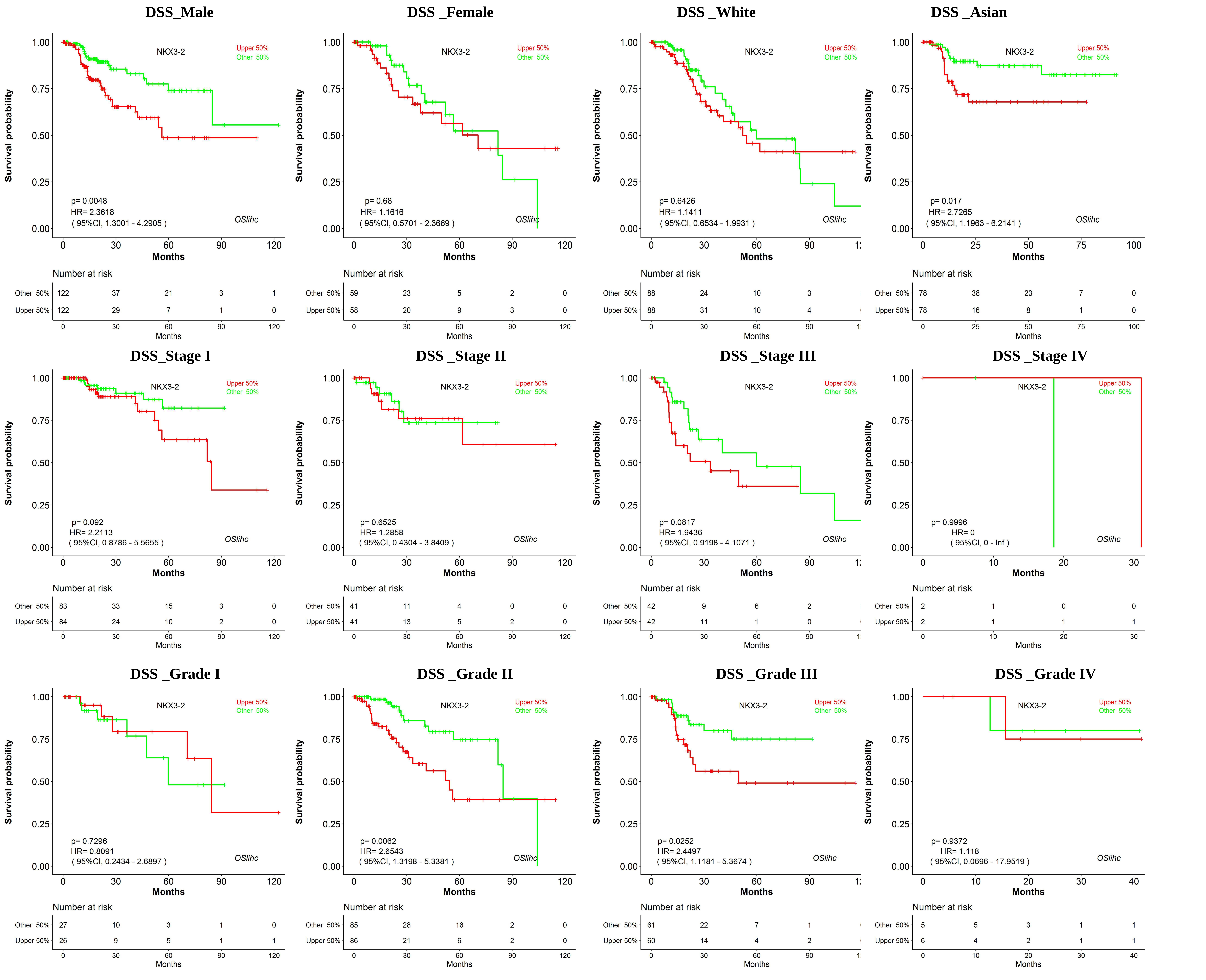

DSS_Male
NKX3-2
Upper 50%
Other 50%
p= 0.0048
HR= 2.3618
(95%CI, 1.3001 - 4.2905)
OSlihc
DSS _Female
p= 0.68
HR= 1.1616
(95%CI, 0.5701 - 2.3669)
DSS _White
p= 0.6426
HR= 1.1411
(95%CI, 0.6534 - 1.9931)
DSS _Asian
p= 0.017
HR= 2.7265
(95%CI, 1.1963 - 6.2141)
DSS_Stage I
p= 0.092
HR= 2.2113
(95%CI, 0.8786 - 5.5655)
DSS _Stage II
p= 0.6525
HR= 1.2858
(95%CI, 0.4304 - 3.8409)
DSS _Stage III
p= 0.0817
HR= 1.9436
(95%CI, 0.9198 - 4.1071)
DSS _Stage IV
p= 0.9996
HR= 0
(95%CI, 0 - Inf)
DSS _Grade I
p= 0.7296
HR= 0.8091
(95%CI, 0.2434 - 2.6897)
DSS _Grade II
p= 0.0062
HR= 2.6543
(95%CI, 1.3198 - 5.3381)
DSS _Grade III
p= 0.0252
HR= 2.4497
(95%CI, 1.1181 - 5.3674)
DSS _Grade IV
p= 0.9372
HR= 1.118
(95%CI, 0.0696 - 17.9519)
Survival probability
Months
Number at risk
